# Multicenter evaluation of a syndromic rapid multiplex PCR test for early adaptation of antimicrobial therapy in adult patients with pneumonia

**DOI:** 10.1186/s13054-020-03114-y

**Published:** 2020-07-14

**Authors:** Céline Monard, Jonathan Pehlivan, Gabriel Auger, Sophie Alviset, Alexy Tran Dinh, Paul Duquaire, Nabil Gastli, Camille d’Humières, Adel Maamar, André Boibieux, Marion Baldeyrou, Julien Loubinoux, Olivier Dauwalder, Vincent Cattoir, Laurence Armand-Lefèvre, Solen Kernéis, the ADAPT study group, Agathe Becker, Julien Charpentier, Julien Textoris, Claude-Alexandre Gustave, Grégory Destras, François Vandenesch, Bruno Lina, Jean Sebastien Casalegno, Manon Lejeune, Philippe Montravers, Claire Poyart, Hugo Tête, Jean-François Timsit, Thomas Uberti

**Affiliations:** 1 Département d’Anesthésie et Réanimation, Hospices Civils de Lyon, Hôpital E. Herriot, Lyon, France; 2 Service de Réanimation Médicale Infectieuse, APHP, Hôpital Bichat Claude Bernard, Paris, France; 3 Service de Bactériologie-Hygiène Hospitalière, CHU de Rennes, Rennes, France; 4 CNR de la Résistance aux Antibiotiques (Laboratoire Associé Entérocoques), Rennes, France; 5 Equipe Mobile d’Infectiologie, APHP, Hôpital Cochin, Centre Université de Paris, Paris, France; 6 Département d’anesthésie-réanimation, APHP, Hôpital Bichat-Claude Bernard, Université de Paris, Paris, France; 7 Inserm U 1148 LVTS, Université de Paris, Paris, France; 8 Service de Bactériologie, APHP, Hôpital Cochin, Centre Université de Paris, Paris, France; 9 Service de Bactériologie, APHP Nord, Université de Paris, Hôpital Bichat, Paris, France; 10 IAME, INSERM, Université de Paris, Paris, France; 11 Service de Maladies Infectieuses et Réanimation Médicale, Hôpital Pontchaillou, Université de Rennes, Rennes, France; 12 Faculté de Médecine, Université de Rennes 1, Unité INSERM CIC 1414, IFR 140, Rennes, France; 13 Equipe mobile d’infectiologie, Hospices Civils de Lyon, Hôpital E. Herriot, Lyon, France; 14 Service de Maladies Infectieuses et Réanimation Médicale, CHU Rennes, Rennes, France; 15 Service de Bactériologie, AP-HP Centre, Hôpital Cochin, Université de Paris, Paris, France; 16 Plateau de Microbiologie 24/24, Institut des Agents Infectieux, Hospices Civils de Lyon, Centre de Biologie et Pathologie Nord, Lyon, France; 17 INSERM CIRI LYON, Equipe “Pathogénie des Staphylocoques”, Lyon, France; 18 CNR de la Résistance aux Antibiotiques (laboratoire associé ‘Entérocoques), Rennes, France; 19 Unité Inserm U1230, Université de Rennes 1, Rennes, France

**Keywords:** Antimicrobials, Antimicrobial stewardship, Pneumonia, Multiplex PCR, Syndromic tests, Biofire® FilmArray®

## Abstract

**Background:**

Improving timeliness of pathogen identification is crucial to allow early adaptation of antibiotic therapy and improve prognosis in patients with pneumonia. We evaluated the relevance of a new syndromic rapid multiplex PCR test (rm-PCR) on respiratory samples to guide empirical antimicrobial therapy in adult patients with community-acquired pneumonia (CAP), hospital-acquired pneumonia (HAP), and ventilator-acquired pneumonia (VAP).

**Methods:**

This retrospective multicenter study was conducted in four French university hospitals. Respiratory samples were obtained from patients with clinical and radiological signs of pneumonia and simultaneously tested using conventional microbiological methods and the rm-PCR. A committee composed of an intensivist, a microbiologist, and an infectious diseases specialist retrospectively assessed all medical files and agreed on the most appropriate antimicrobial therapy for each pneumonia episode, according to the results of rm-PCR and blinded to the culture results. The rm-PCR-guided antimicrobial regimen was compared to the empirical treatment routinely administered to the patient in standard care.

**Results:**

We included 159 pneumonia episodes. Most patients were hospitalized in intensive care units (*n* = 129, 81%), and episodes were HAP (*n* = 68, 43%), CAP (*n* = 54, 34%), and VAP (*n* = 37, 23%). Conventional culture isolated ≥ 1 microorganism(s) at significant level in 95 (60%) patients. The syndromic rm-PCR detected at least one bacteria in 132 (83%) episodes. Based on the results of the rm-PCR, the multidisciplinary committee proposed a modification of the empirical therapy in 123 (77%) pneumonia episodes. The modification was a de-escalation in 63 (40%), an escalation in 35 (22%), and undetermined in 25 (16%) patients. In microbiologically documented episodes (*n* = 95), the rm-PCR increased appropriateness of the empirical therapy to 83 (87%), as compared to 73 (77%) in routine care.

**Conclusions:**

Use of a syndromic rm-PCR test has the potential to reduce unnecessary antimicrobial exposure and increase the appropriateness of empirical antibiotic therapy in adult patients with pneumonia.

## Background

Inadequate and delayed empirical treatments are strong predictors of mortality in sepsis [1, 2]. Therefore, in pneumonia patients, international guidelines state that an attempt should be made to obtain respiratory samples and recommend to start early empirical treatment while awaiting for the results of culture and antimicrobial susceptibility testing (AST) [3]. For severe patients or those with risk factors of multidrug-resistant organisms (MDRO), the empirical treatment should include a broad-spectrum antibiotic [4, 5]. However, conventional microbiological techniques have a low sensitivity, particularly on microbiological samples collected in non-intubated patients and in case of prior exposure to antibiotics [6, 7]. The lack of a reliable microbiological diagnosis thus prevents from de-escalating the empirical regimen in a large proportion of patients [8].

Identification of causative microorganisms provides the potential to target antibiotic therapy, but the turnaround time from microbiological sampling to AST usually requires at least 48 h. New molecular diagnostic tools aim at shortening this time. Syndromic rapid multiplex PCR (rm-PCR) can be used for simultaneous detection of multiple organisms and resistance markers in a specific clinical context, within a few hours [9]. Alongside with antimicrobial stewardship (AMS), the use of rm-PCR has been shown to significantly decrease time-to-appropriate therapy and optimize clinical and economic outcomes [10, 11]. During a previous evaluation in community-acquired pneumonia, a rm-PCR assay achieved pathogen detection in 87% of patients compared to 39% using culture-based methods [12]. In addition, in this population, molecular testing had the potential to lead to a de-escalation in the number and/or spectrum of initial empirical antibiotics in 77% of patients.

**Fig. 1:**
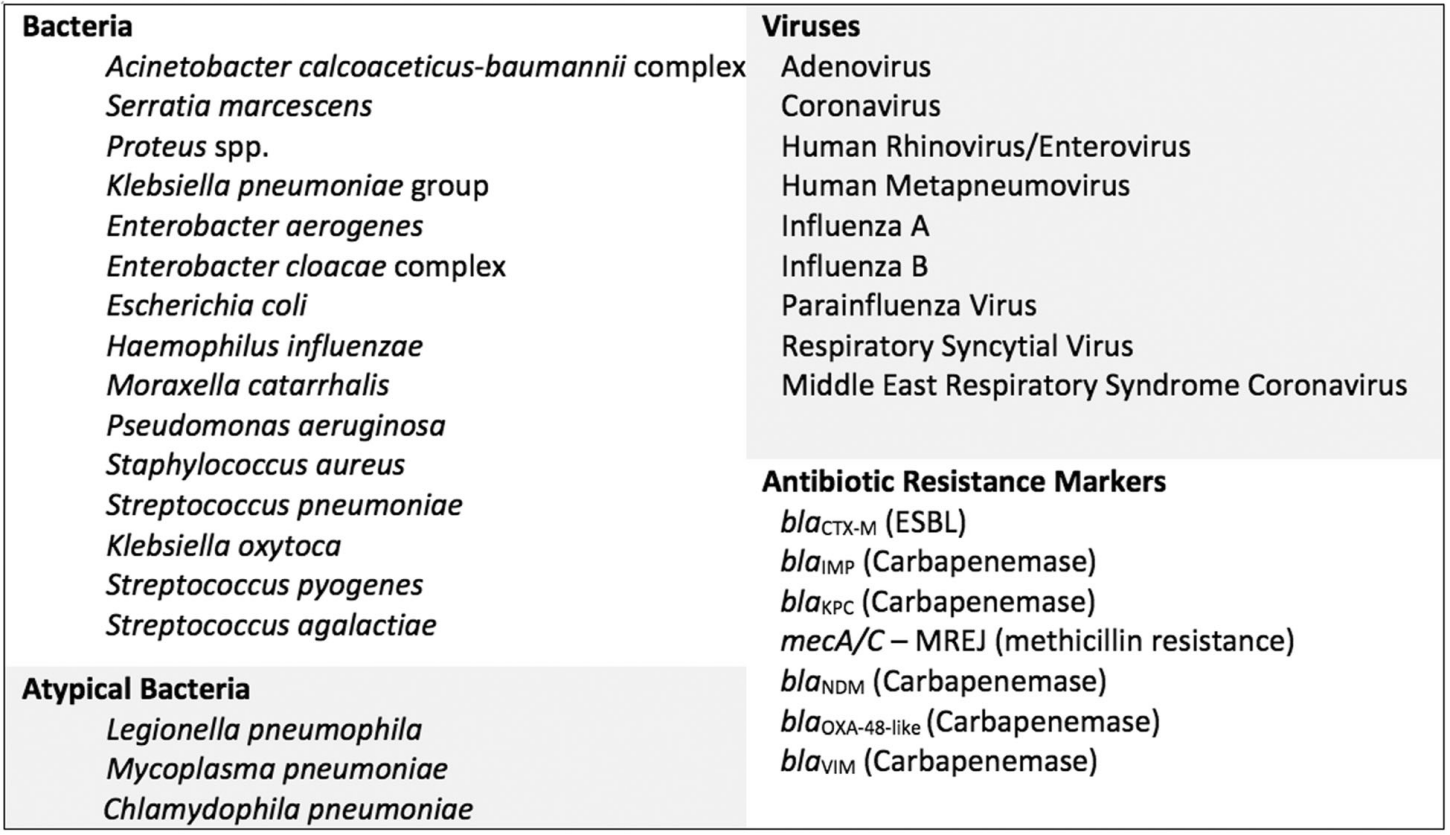
Pathogens targeted by the BioFire® FilmArray® Pneumonia Panel

The BioFire® FilmArray® Pneumonia Panel (bioMerieux S.A., Marcy-l’Etoile, France) is a novel assay able to simultaneously identify 27 of the most common pathogens involved in lower respiratory tract infections (semi-quantitative results for 11 Gram-negative and 4 Gram-positive bacteria, qualitative results for 3 atypical bacteria and 9 viruses) as well as 7 antibiotic resistance genes (Fig. 1). Two studies have found excellent agreement between this molecular method and standard culture [13, 14].

Our main objective was to estimate the potential impact of this new syndromic rm-PCR assay on early adaptation of empirical antimicrobial therapy in adult patients with pneumonia.

## Methods

### Settings and participants

Between July and December 2018, 11 French university hospitals participated in a pre-commercialization evaluation of the investigative-use-only (IUO) version of the rm-PCR BioFire® FilmArray® Pneumonia Panel (bioMerieux S.A., Marcy-l’Etoile, France). A total of 515 respiratory samples (sputa, endotracheal aspirations (ETA), blind bronchial sampling (BBS), and bronchoalveolar lavages (BAL)) were tested simultaneously, using conventional techniques and the rm-PCR. The syndromic rm-PCR demonstrated high agreement with conventional culture in this panel of patients [14, 15].

Four centers (Bichat-Paris, Cochin-Paris, Rennes, and Lyon) were further selected to participate in the present sub-study. Criteria for patient inclusion were as follows: (1) age ≥ 18 years and (2) presence of clinical and radiological criteria for pneumonia according to the IDSA guidelines: new lung infiltrate on a chest X-ray and evidence that the infiltrate was of an infectious origin, i.e., at least two of three clinical features (fever greater than 38 °C, leukocytosis or leukopenia, and purulent secretions) [16]. Criteria for pneumonia were evaluated by two clinical investigators in each center. Community-acquired pneumonia (CAP), hospital-acquired pneumonia (HAP), and ventilator-associated pneumonia (VAP) were included. HAP was defined as pneumonia occurring 48 h or more after admission, which was not incubating at the time of admission and not associated with mechanical ventilation [4]. VAP referred to pneumonia arising > 48 h after endotracheal intubation [4, 16]. CAP included all episodes of pneumonia acquired outside of the hospital setting. Even if not strictly included in the most recent definition of CAP [17], we also included patients with immunocompromising conditions.

### Microbiology methods

Respiratory specimens were routinely analyzed in each local microbiology laboratory according to current recommendations of the French Standard Guidelines in Medical Microbiology (REMIC) [18]. In brief, sputum and ETA samples were digested and diluted according to sample types. Then, they were streaked on recommended plates according to semi-quantitative patterns and incubated for 2 days in CO_2_ and aerobic conditions. Results of standard culture were expressed in colony-forming unit (CFU)/mL. The thresholds for positivity on culture were ≥ 10^3^ CFU/mL for BBS, ≥ 10^4^ CFU/mL for BAL, ≥ 10^5^ CFU/mL for ETA, and ≥ 10^7^ CFU/mL for sputa [19]. Diagnostic tests for viruses and atypical bacteria were conducted only if requested by the physician in charge.

Respiratory samples were simultaneously tested with the IUO version of the BioFire® FilmArray® Pneumonia Panel according to the manufacturer’s instructions directly on native respiratory samples. The IUO version is identical to the final FDA-cleared and CE-marked version. The system integrates sample preparation, nucleic acid extraction and purification, amplification, detection, and analysis, with a total run time of about 1 h. Results of the syndromic rm-PCR are expressed as semi-quantitative results (10^4^ to ≥ 10^7^) in DNA-copies/mL for commonly culturable bacteria and as qualitative results (presence/absence) for resistance genes, viruses, and atypical bacteria. Bacteria found under the threshold of 10^3.5^ copies/mL are not reported on the final rm-PCR report.

### Data sources

Clinical and demographical data were retrospectively obtained from the electronic medical records of each patient. Investigators collected demographic characteristics (age, gender) and medical data such as medical history, classification of pneumonia (VAP, HAP, or CAP), severity scores, and antibiotics prescribed.

In each study center, a multidisciplinary committee composed of an intensivist, an infectious diseases specialist, and a clinical microbiologist was formed. During a face-to-face meeting, the local multidisciplinary committee retrospectively reviewed medical files of all pneumonia patients, including patients’ medical history, previous antimicrobial treatments received, previous microbial colonization and risk factors for MDRO carriage, clinical parameters (e.g., fever, hemodynamics, and respiratory parameters), results of standard biological analyses, and chest imaging. For each episode, the result of the syndromic rm-PCR was presented to the committee, blinded to (1) the direct examination of the sample, (2) the results of the standard culture and AST, and (3) the empirical therapy administered in routine care. Guided by the rm-PCR results, the multidisciplinary committee agreed on the most appropriate antimicrobial therapy for each pneumonia episode.

### Endpoints

The antimicrobial therapy proposed by the multidisciplinary committee based on syndromic rm-PCR results (PCR-guided therapy) was compared to the empirical therapy actually delivered to the patient in routine care (empirical therapy). The primary endpoint was the number of pneumonia episodes in which PCR-guided therapy differed from empirical therapy. The secondary endpoints were as follows: (1) the number of de-escalations of the antibiotic regimen based on syndromic rm-PCR results and (2) the number of episodes in which PCR-guided therapy would be active on pathogens isolated at significant threshold on culture. Both primary and secondary endpoints were evaluated by an independent central committee composed of an intensivist and an infectious disease specialist from two different university hospitals (SK and CM).

**Fig. 2:**
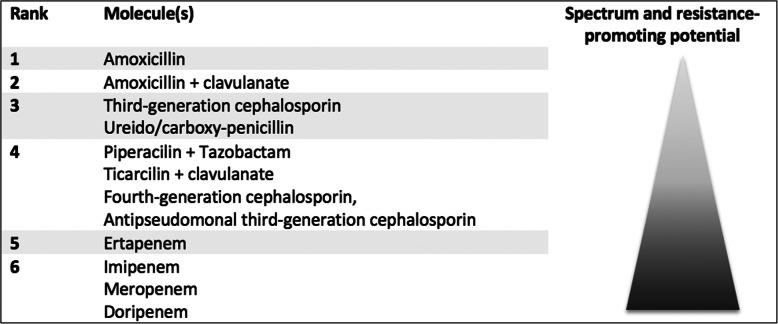
Consensual ranking of *β*-lactams according to both their spectrum and their resistance-promoting potential according to Weiss et al., *Clinical Microbiology and Infection* 2015 “Elaboration of a Consensual Definition of De-Escalation Allowing a Ranking of β-Lactams”

De-escalation was defined according to previous studies as discontinuation of any companion drug and/or switch of the pivotal drug to a narrower-spectrum antibiotic [20, 21]. Ranking of pivotal drugs was determined according to their antibacterial spectrum and putative ecological impact and defined either within the same antibiotic family or from a family to another (e.g., vancomycin to oxacillin). β-lactams were categorized into six groups, as previously described by Weiss et al. [22] (Fig. 2). In several cases (e.g., switch from piperacillin/tazobactam to a fourth-generation cephalosporin), as hierarchy of antibiotics could not be established, the antibiotic change was qualified as “undetermined.” If a companion drug was stopped but a new one was started, the antibiotic change was also considered as “undetermined.”

Definitive results of standard culture and AST were considered by the central committee to state on the susceptibility of pathogens to the PCR-guided therapy. This secondary endpoint was evaluated only in microbiologically documented pneumonia episodes. Only pathogenic microorganisms cultured at significant levels on culture were considered as documentation. Coagulase-negative staphylococci (CoNS) and *Enterococcus* spp. were considered as non-pathogenic.

### Statistical methods and ethics

All data were anonymously collected and stored on a secured database. Descriptive analysis was performed using the R software (3.3.2, R Foundation for Statistical Computing, Vienna, Austria). Due to relatively small numbers and the retrospective design of our study, we did not perform a formal statistical analysis, but rather present the description of data. For each variable, when data were missing, they were excluded from the calculation of percentages. According to the French legislation, this study was approved by the Ethics Committee of the French Society of Infectious Diseases (*Société de Pathologie Infectieuse de Langue Française*), n°2019-0902, and declared to the French national data protection commission (*Commission Nationale de l’Informatique et des Libertés*, *CNIL*), n°19-213.

## Results

### Patients

Among 170 pneumonia episodes included in the pre-commercialization evaluation in the four investigating centers, 159 were retained for final analysis. Exclusion criteria were as follows: age < 18 years (*n* = 2) and criteria for pneumonia not fulfilled (*n* = 9). A total of 129 patients (81%) were hospitalized in intensive care units (ICUs) at inclusion, and their median [IQR] SOFA score was 6 [2.5–8.5]. Overall in-hospital mortality was 28% (45/159). Pneumonia episodes were classified as HAP (*n* = 68, 43%), CAP (*n* = 54, 34%), and VAP (*n* = 37, 23%). Microbiological samples were as follows: ETA (*n* = 71, 45%), sputum samples (*n* = 33, 21%), BAL (*n* = 34, 21%), and BBS (*n* = 21, 13%). Other demographic data are reported in Table 1.

**Table 1:** Characteristics of study participants (*n* = 159)

	Overall *n* = 159	CAP *n* = 54	HAP *n* = 68	VAP *n* = 37
**Clinical features**				
Admission ward				
Intensive care unit	129 (81)	41 (76)	51 (75)	37 (100)
Surgical unit	13 (8)	1	12 (18)	–
Medical unit	12 (7)	7 (13)	5 (7)	–
Emergency room	5 (4)	5 (9)	–	–
Demographics				
Age, years	63 [55–72]	64 [55–74]	64 [56–71]	61 [53–69]
Male	107 (67)	32 (59)	46 (67)	29 (78)
Comorbid conditions				
Immune suppression^a^	52 (33)	11 (20)	29 (43)	12 (32)
Chronic pulmonary conditions^b^	52 (33)	19 (35)	26 (38)	7 (19)
Charlson score	2 [1–5]	2 [1–4]	3 [1–6]	2 [1–5]
Severity of disease at inclusion				
SOFA score (in ICU patients)	6 [4–9]	6 [4–9]	6 [4–9]	6 [4–8]
Septic shock	49 (31)	13 (24)	18 (27)	18 (49)
Initiation of respiratory support	64 (40)	20 (37)	34 (50)	–
In-hospital death	45 (28)	14 (26)	13 (19)	18 (49)
**Type of microbiological sampling**				
Endotracheal aspiration	71 (45)	30 (55)	33 (49)	8 (22)
Sputum	33 (21)	13 (24)	20 (29)	–
Bronchoalveolar lavage	34 (21)	4 (7)	13 (19)	17 (46)
Blind bronchial sampling	21 (13)	7 (13)	2 (3)	12 (32)
**Microbiology—conventional techniques**				
*Escherichia coli*	17 (11)	4 (7)	5 (7)	8 (22)
*Pseudomonas aeruginosa*	15 (9)	3 (6)	8 (12)	4 (11)
*Staphylococcus aureus*	15 (9)	3 (6)	4 (6)	8 (22)
*Klebsiella pneumoniae group*	10 (6)	2 (4)	5 (7)	3 (8)
*Haemophilus influenzae*	10 (6)	4 (7)	4 (6)	2 (5)
*Streptococcus pneumoniae*	6 (4)	3 (6)	3 (4)	–
*Enterobacter cloacae complex*	7 (4)	1	5 (7)	1 (3)
*Enterobacter aerogenes*	4 (3)	1	2 (3)	1
*Citrobacter freundii*	2 (1)	–	2 (3)	–
*Serratia marcescens*	2 (1)	–	1	1
*Morganella morganii*	2 (1)	–	–	2 (5)
*Raoultella*	2 (1)	1	–	1
*Stenotrophomonas maltophilia*	2 (1)	–	–	2 (5)
*Acinetobacter calcoaceticus-baumannii complex*	1	–	–	1
*Legionella pneumophila*	1	1	–	–

Results are presented as *n* (%) or median [interquartile range]

Conventional techniques include culture for all pathogens presented, apart from *Legionella pneumophila* which was detected using molecular techniques*.* Only pathogens cultured at pre-defined levels are presented (≥ 10^3^ CFU/ml for blind bronchial sampling, ≥ 10^4^ CFU/ml for bronchoalveolar lavage, ≥ 10^5^ CFU/ml for endotracheal aspiration, and ≥ 10^7^ CFU/ml for sputum samples)

^a^Immunosuppression was defined as leucopenia < 1 Giga/L, HIV infection, history of solid organ or stem cell transplantation, and immunosuppressive treatment including corticosteroids over 10 mg/day of equivalent prednisone for more than 15 days

^b^Chronic pulmonary condition included patients with history of chronic obstructive pulmonary disease, severe bronchiectasis, cystic fibrosis, and chronic respiratory insufficiency

### Microbiology and routine antimicrobial therapy

Conventional culture isolated ≥ 1 microorganism(s) at significant level in 95 episodes (60%): 27/54 (50%) in CAP, 40/68 (59%) in HAP, and 28/37 (76%) in VAP. The syndromic rm-PCR detected at least one bacteria in 132 (83%) episodes; a viral co-infection was detected in 16 episodes and a virus without bacteria in 6 episodes. Among the 64 undocumented episodes on culture, the syndromic rm-PCR identified a microorganism in 21 episodes (bacteria in 19 episodes and viruses in 2 episodes). The main pathogens identified on culture were Gram-negative bacilli (*Escherichia coli* (24), *Pseudomonas aeruginosa* (23), *Enterobacter cloacae* complex (14), *Klebsiella pneumoniae* (12), *Haemophilus influenzae* (11)) and Gram-positive cocci (*Staphylococcus aureus* (28), *Streptococcus pneumoniae* (8)) (Table 2). Results of the microbiological analyses according to sample type are described in the supplementary material (Supplementary Table 1). Multiple microorganisms were detected in 33% of episodes using culture and 43% using the syndromic rm-PCR method. No influenza virus was found, neither using standard methods nor the syndromic rm-PCR. The syndromic rm-PCR identified 16 antibiotic resistance genes: 11 *bla*_CTX-M_ and 5 *mecA/C*- MREJ genes. All pathogens identified as harboring an extended-spectrum β-lactamase on AST (*n* = 10) were found positive for the *bla*_CTX-M_ by the rm-PCR. The 2 *Staphylococcus aureus* strains resistant to methicillin on AST were detected by the rm-PCR, which detected 2 more methicillin-resistant *Staphylococcus aureus* strains that were not found on culture. Six Enterobacteriaceae probably had an overproduction of AmpC β-lactamase, which could not be detected by the rm-PCR.

The empirical therapy included a β-lactam in 147 (92%) pneumonia episodes: third-generation cephalosporin (*n* = 35, 22%), amoxicillin/clavulanate (*n* = 27, 17%), piperacillin/tazobactam (*n* = 29, 18%), or fourth-generation cephalosporin (*n* = 19, 12%), and carbapenems (*n* = 27, 17%). A companion molecule was prescribed in 79 (50%) episodes: mainly macrolides (*n* = 20, 13%), imidazoles (*n* = 17, 11%), and aminoglycosides (*n* = 15, 9%; Table 3).

**Table 2:** Comparison of microbiological documentation obtained by conventional techniques versus rm-PCR in 159 pneumonia episodes

Pathogen identification	Conventional techniques	rm-PCR
**Targets included in the rm-PCR panel**		
Gram-negative bacilli		
*Escherichia coli*	24	33
*Pseudomonas aeruginosa*	23	28
*Enterobacter cloacae complex*	13	22
*Klebsiella pneumoniae group*	13	14
*Haemophilus influenzae*	11	33
*Acinetobacter calcoaceticus-baumannii complex*	3	4
*Enterobacter aerogenes*	5	7
*Serratia marcescens*	3	5
*Proteus spp.*	2	8
*Moraxella catarrhalis*	1	5
*Klebsiella oxytoca*	1	5
Gram-positive cocci		
*Staphylococcus aureus*	26	42
*Streptococcus pneumoniae*	8	16
*Streptococcus pyogenes*	0	0
*Streptococcus agalactiae*	0	0
Atypical bacteria		
*Legionella pneumophila*	1	1
*Mycoplasma pneumoniae*	0	1
*Chlamydophila pneumoniae*	0	0
Resistance		
mecA		5
CTX-M		11
**Targets not included in the rm-PCR panel**		
*Stenotrophomonas maltophilia*	4	–
*Morganella morganii*	3	–
*Raoultella ornithinolytica*	3	–
*Citrobacter sp.*	4	–
*Hafnia*	1	
Others*	11	–

Conventional techniques include culture for all pathogens presented, apart from *Legionella pneumophila* which was detected using molecular techniques

*rm-PCR* real-time multiplex polymerase chain reaction

*Acinetobacter junii (*n* = 1), *Actinomyces odontolyticus* (*n* = 1), *Corynebacterium* sp. (*n* = 1), coagulase-negative *Staphylococcus* (*n* = 4), *Enterococcus faecium* (*n* = 1), *Enterococcus faecalis* (*n* = 6), *Lactobacillus reuteri* (*n* = 1), *Streptococcus alpha* (*n* = 1), *Streptococcus pseudopneumoniae* (*n* = 1)

**Table 3:** Comparison of antimicrobial therapies prescribed in the empirical and the rm-PCR-guided groups (*n* = 159)

Antibiotic treatment	Routine empirical therapy (%)	rm-PCR-guided therapy (%)
Beta-lactams	147 (92)	131 (82)
Third-generation cephalosporin	35 (22)	24 (15)
Piperacillin-tazobactam	29 (18)	34 (21)
Amoxicillin-clavulanate	27 (17)	25 (16)
Carbapenem	27 (17)	19 (12)
Cefepime	19 (12)	19 (12)
Amoxicillin	5 (3)	4 (3)
Ceftazidime	3 (2)	7 (4)
Cefazolin	2 (1)	6 (4)
Penicillin M	0 (0)	2 (1)
Companion molecule	79 (50)	50 (31)
Macrolide	20 (13)	4 (3)
Metronidazole	17 (11)	8 (5)
Aminoglycoside	15 (9)	19 (12)
Fluoroquinolone	8 (5)	5 (3)
Glycopeptide	6 (4)	2 (1)
Linezolide	6 (4)	8 (5)
Cotrimoxazole	7 (4)	4 (3)
Antifungal therapy	5 (3)	4 (3)
Oseltamivir	3 (2)	0
Other	4 (3)	0

Results are presented as *n* (%)

### Impact of the syndromic rm-PCR on antimicrobial therapy

Based on the results of the rm-PCR, the multidisciplinary committee proposed a modification of the routine empirical therapy in 123 (77%) pneumonia episodes: de-escalation in 63 (40%) and escalation in 35 (22%; Table 4). The proportion of antibiotic modifications was higher in VAP (32/37, 87%), as compared to HAP (54/68, 79%) and CAP (37/54, 69%). De-escalation consisted in change of the pivotal drug to a narrower spectrum antibiotic (*n* = 37, 23%) and/or discontinuation of a companion drug (*n* = 37, 23%), or discontinuation of all antibiotics (*n* = 9, 6%). Sputum samples were associated with less antimicrobial modifications (21/33, 64%) compared to other sample types (58/71, 82%; 27/34, 80%; 17/21, 81%; for ETA, BAL, and BBS respectively). Modifications in the sputum group were escalation and de-escalation in the same number of cases, whereas there was more de-escalation compared to escalation in the other type of samples (Supplementary Table 2).

**Table 4:** Impact of the rm-PCR results on antibiotic prescription, according to the multidisciplinary committee (*n* = 159)

	Overall, *n* = 159	CAP, *n* = 54	HAP, *n* = 68	VAP, *n* = 37
Antibiotic modification	123 (77)	37 (69)	54 (79)	32 (87)
De-escalation	63 (40)	20 (37)	25 (37)	18 (49)
Escalation	35 (22)	8 (15)	18 (27)	9 (24)
Undetermined	25 (16)	9 (17)	11 (16)	5 (14)
No change	36 (23)	17 (32)	14 (21)	5 (14)

Results are presented as *n* (%)

*CAP* community-acquired pneumonia, *HAP* hospital-acquired pneumonia, *VAP* ventilator-associated pneumonia

In microbiologically documented episodes (*n* = 95), pathogens were susceptible to the empirical therapy in 73 (77%) episodes and to the PCR-guided therapy in 83 (87%) episodes. The proportion of the empirical and PCR-guided therapies that were active on the documented pathogens did not statistically differ between CAP, HAP, and VAP (data not shown). When the PCR-guided therapy differed from the empirical therapy, pathogens were susceptible to PCR-guided therapy in 85% [28/33] of the de-escalations and 88% [22/25] of the escalations. For 14 (9%) patients, the PCR-guided therapy was active against documented pathogens, whereas the empirical routine therapy was non-active. Conversely, 4 patients had an active empirical therapy, and the multidisciplinary committee proposed a non-active PCR-guided therapy. In 1 patient, the syndromic rm-PCR was negative and the committee proposed to discontinue antibiotics, whereas standard culture grew up 10^7^ CFU/ml *Morganella morganii* (not included in the panel). Of note, Gram-negative bacilli were identified on direct examination of the respiratory sample (BAL), but this information was not considered by the multidisciplinary committee. In 2 other patients treated with fourth-generation cephalosporin as empirical therapy, the committee proposed a treatment with piperacillin-tazobactam. These 2 patients had a syndromic rm-PCR positive for *Enterobacter cloacae complex* confirmed by culture, consequently found resistant to piperacillin-tazobactam due to overexpression of the naturally produced AmpC β-lactamase. The fourth patient was empirically treated with ceftolozane-tazobactam, and the committee proposed to treat him with ceftazidime. Both syndromic rm-PCR and conventional culture identified *Pseudomonas aeruginosa*, which was phenotypically confirmed as resistant to ceftazidime. Of note, this patient was already known to be colonized with a *P. aeruginosa* of this particular phenotype. Details on all inadequate antibiotic therapies (either empirical or PCR-guided) are available in the supplementary material (Supplementary Table 3).

Overall, as described in Table 3, compared to routine empirical therapy, the syndromic rm-PCR would have theoretically led to avoid 27 prescriptions of β-lactams (third-generation cephalosporins [*n* = 15], carbapenems [*n* = 8], amoxicillin-clavulanate [*n* = 2]), 29 prescriptions of companion antibiotics (macrolides, metronidazole, fluoroquinolones, glycopeptides, cotrimoxazole), and 8 other molecules (antifungal therapy, oseltamivir, other beta-lactams). It would however have increased the number of prescriptions of piperacillin-tazobactam (*n* = 5), ceftazidime (*n* = 4), cefazolin (*n* = 4), and penicillin M (*n* = 2).

## Discussion

In this retrospective multicenter study, a syndromic rm-PCR approach with semi-quantitative results had the potential to lead to a change in empirical antimicrobial therapy in 77% of pneumonia episodes in adult patients. The most frequent intervention was a de-escalation, which occurred in almost half of the patients. Using standard culture and AST as the reference method, PCR-guided antibiotic treatment was more frequently adequate when compared to the empirical treatment.

As observed in previous studies, microbiological documentation was almost twice as high using the rm-PCR compared to the standard method [14]. If this higher sensitivity is an advantage for patients treated with antibiotics prior to sampling, it also implies a very cautious interpretation of results. The test might detect nucleic acids from dead pathogens that are not involved in the actual pneumonia episode. As there is no scientific data regarding the clearance of bacterial genomic material from the lung, this could lead to overtreatment of non-viable microorganisms. Similarly, distinguishing colonization from infection is challenging and requires interpreting the results in accordance with the clinical context. Even if semi-quantitative quantification of bacterial targets provided by the BioFire® FilmArray® Pneumonia Panel is helpful to guide the decision, there are currently no consensual thresholds of clinical significance for molecular methods. In the present report, we did not evaluate the correlation between culture quantification (in CFU/ml) and molecular semi-quantification (in DNA copies/ml). This question is currently under investigation [15]. Meanwhile, clinicians should be aware that molecular diagnosis is highly sensitive and the use of thresholds established for conventional culture to guide interpretation of the rm-PCR is not straightforward and may lead to misinterpretation.

It should also be kept in mind that even if it detects a large panel of microorganisms, the test is not exhaustive and several important pathogens (such as *Morganella* spp., *Citrobacter* spp., *Hafnia alvei*, *Stenotrophomonas maltophilia*, or *Pneumocystis jirovecii*) are not included. The same issue can be raised for the resistance genes, especially ESBL-encoding genes for which only the most frequent ones (i.e., *bla*_CTX-M_) are included in the panel. Thus, when a pathogen is identified by the test, the absence of resistance genes does not predict with certainty its phenotypic susceptibility. When a gene encoding for a resistance marker is detected alongside with different bacteria, it is not possible to link the resistance to one of the detected microorganisms. Also, overexpression of intrinsic resistance genes (such as AmpC β-lactamase) is not detected by the panel. From this perspective, rm-PCR should not be considered as a sufficient diagnostic test for pneumonia but rather as a complementary tool in adjunction to standard culture and AST, to allow for an earlier pathogen-directed therapy. Multidisciplinary discussion of the results is a prerequisite for optimal use. In our experience, the rare errors (4 over 159 episodes) in the choice of PCR-guided therapy were linked to (1) Amp-C overproduction in *Enterobacter* sp. in 2 cases, (2) isolation of pathogens not included in the panel (i.e., *Morganella morganii*) in 1 case, and (3) isolation of a multi-drug-resistant *P. aeruginosa* (that was previously isolated in this particular patient) in 1 case. In this last case, it is important to note that interpretation of the rm-PCR in light of the previous microbial documentation could have prevented this error.

Our intervention was based on a multidisciplinary approach with physicians from different specialties interpreting the results of the rm-PCR together and agreeing on an optimal therapy. The success of this approach is concordant with previous studies where strategies combining rapid diagnostic testing and AMS interventions in bloodstream infections showed a significant and synergistic impact on early antibiotic de-escalation, mortality, and costs [23–26]. Moreover, implementation of AMS programs, led by dedicated multidisciplinary teams, is strongly encouraged to improve the quality of antibiotic prescriptions [27–29]. Interestingly, the use of rapid diagnostic tests without active AMS intervention failed to provide the expected benefits in patients [26, 30–32]. It is uncertain whether the same results would have been observed if only one clinician, non-expert of the diagnostic methods, had to choose alone a therapy guided by the rm-PCR results. Another strength of the present study is its multicenter character, as it improves the generalizability of the results, but also because the multidisciplinary committees were local and therefore aware of the ecology of their hospitals.

The present study presents some limitations. Its retrospective and observational design did not allow comparison of clinical outcomes with the rm-PCR versus standard treatment approach. Second, although we included a large panel of pneumonia patients (severe ICU patients with VAP or HAP and non-severe CAP patients from the emergency room), it remains unclear what group of patients will benefit most from the syndromic rm-PCR diagnosis. Further studies should evaluate this new test in more selected groups of patients. However, most patients herein were severe patients hospitalized in ICUs with complicated microbiological histories, and results suggest the rm-PCR could help to choose the antimicrobial treatment even in this particular group of patients. Also, even if we tried to give the multidisciplinary committee as much information as needed to choose the antibiotic treatment, in some cases, important data may have been missing. As an example, this might explain why the clinician chose a ceftolozane-tazobactam-based treatment for a patient with a past history of infection with piperacillin-tazobactam-resistant *P. aeruginosa*, whereas the committee decided to use piperacillin-tazobactam despite identification of *P. aeruginosa* by the rm-PCR. Again, this reinforces the necessity to carefully interpret the rm-PCR report, in light of the clinical and historical microbiological data. In our panel of CAP patients, only half had microbiological documentation on conventional culture. This is in accordance with previous studies that highlighted a low rate of documentation in CAP, due to difficulties to obtain contributive microbiological samples and to previous exposure to antibiotics [6]. In the 27 CAP patients with positive microbiology results, we identified 10 pathogens frequently involved in CAP (*Staphylococcus aureus*, *Haemophilus influenzae*, *Streptococcus pneumoniae*) and 12 Gram-negative bacilli (4 *Escherichia coli*, 3 *Pseudomonas aeruginosa*, 2 *Klebsiella pneumoniae*, 2 *Enterobacter* sp., 1 *Raoultella*). The number of Gram-negative bacteria isolated is likely related to the profile of patients included in our study. Indeed, all participating centers were tertiary university hospitals, referral centers for specific comorbidities as highlighted in Table 1: among CAP patients, 35% had chronic respiratory conditions and 20% immune suppression.

The gold standard used herein for microbiological diagnosis was culture with AST. In certain cases, the syndromic rm-PCR detected pathogens that were not detected by culture. This finding questions the choice of culture as the gold standard for microbial identification, notably in case of antibiotic treatment before sampling. Because the molecular diagnostic methods are probably more sensitive than cultures, the present results would have been even more favorable for PCR-guided therapy if we had also considered as documentation the organisms identified by rm-PCR and not by culture. As an example, one patient had a *Staphylococcus aureus* (10^5^ copies/mL), a *Moraxella catarrhalis* (10^4^ copies/mL), and a mecA/C-MREJ gene found by rm-PCR while the culture remained negative and was therefore considered as “undocumented.” Also, because two thirds of our samples were non-protected (ETA and sputum), this could have led to an over-identification of bacteria [33]. However, since we used the culture and validated the significance thresholds for interpretation, this bias was minimized. The high proportion of non-invasive samples reflects the heterogeneity of practices between ICUs and is consistent with the IDSA guidelines which suggest noninvasive sampling with semi-quantitative cultures to diagnose VAP, rather than invasive sampling [4].

Many questions regarding the syndromic rm-PCR still remain unanswered and should be addressed in future evaluations. The cost-effectiveness ratio of the syndromic rm-PCR needs to be evaluated in order to justify its use in particular subgroups of patients. To date, no studies addressed this medico-economic outcome in lower respiratory tract infections, but a syndromic rm-PCR developed for the higher tract respiratory infections was found to decrease the length of hospital stay, duration of antibiotic therapy, and time in isolation, therefore decreasing related costs [34, 35]. As previously discussed, thresholds for interpretation of rm-PCR results cannot be directly derived from those established for culture and must be more precisely defined. Because of these limits, physicians should be aware of the test’s drawbacks and the present results highlight the need for a close cooperation between infectious disease specialists, clinical microbiologists, and clinicians for the interpretation of results. We also suggest that the results of the test should be included in a decision algorithm, alongside with the suspected pathogens, the history of the patient, and their personal risk factors for MDRO infection. We recommend that decision regarding antibiotic treatment based on rm-PCR results should consider clinical presentation, local epidemiology, direct smear of the sample, and historical microbiological data in each patient. As a guidance for use in clinical practice and based on our experience, we may recommend to (1) consider the semi-quantitative result regarding the type of sample, in order to discriminate between colonization and infection (future results should be available soon to precise the significance thresholds); (2) if a microorganism is identified and thought to be pathogenic, the antimicrobial therapy should be chosen considering colonization of the patient, previous microbial documentations, and risk factors for MDRO; (3) if a pathogen of the group 3 is detected, an Amp-C overproduction cannot be excluded and therefore treatment with cefepim or meropenem should be considered; (4) oseltamivir should not be prescribed in the absence of influenza detection; (5) a companion molecule such as macrolide could be discontinued for community-acquired pneumonia without identification of atypical bacteria; and (6) identification of a resistance gene should always be considered.

To date, the rm-PCR should not be considered as a sufficient diagnostic test for pneumonia but rather as a complementary tool in adjunction to standard culture and AST, to allow for an earlier pathogen-directed therapy. Controlled randomized trials evaluating the impact of rm-PCR on patient’s outcomes are currently ongoing, and their results will be of major help to precise the impact of the rm-PCR on infection control, length of stay, and resistance selection.

## Conclusion

Results suggest that early use of rm-PCR in pneumonia could reduce unnecessary antimicrobial exposure without compromising the appropriateness of the treatment. Together with an expert advice, this promising diagnostic tool could improve the quality of care and participate in the reduction of broad-spectrum antimicrobial agents’ use. Prospective randomized controlled trials are needed to confirm these results, identify categories of patients that would most benefit from the test, and define precise guidelines to appropriately adjust the empirical therapy based on the results.

## Supplementary information

**Additional file 1: Supplementary table 1.** Microbiological documentation depending on the sample type (*n*=159). **Supplementary table 2.** Impact of the rm-PCR results on antibiotic prescription, according to the multidisciplinary committee and regarding sample type (n=159). **Supplementary Table 3.** Description of inadequate antibiotic regimen.

## Data Availability

The datasets used during the current study are available from the corresponding author on reasonable request.

## Abbreviations

AMS: Antimicrobial stewardship program
AST: Antimicrobial susceptibility testing
BAL: Bronchoalveolar lavage
CAP: Community-acquired pneumonia
HAP: Hospital-acquired pneumonia
ICU: Intensive care unit
MDRO: Multidrug-resistant organism
rm-PCR: Real-time multiplex PCR
VAP: Ventilator-acquired pneumonia
