# Supplementary material for: Multicenter evaluation of a syndromic rapid multiplex PCR test for early adaptation of antimicrobial therapy in adult patients with pneumonia
Source: Crit Care. 2020 Jul 14;24:434. doi: 10.1186/s13054-020-03114-y (PMC7359443; doi:10.1186/s13054-020-03114-y)
Supplement: Supplementary file 1 — Additional file 1: Supplementary table 1. Microbiological documentation depending on the sample type (n=159). Supplementary table 2. Impact of the rm-PCR results on antibiotic prescription, according to the multidisciplinary committee and regarding sample type (n=159). Supplementary Table 3. Description of inadequate antibiotic regimen. [file 13054_2020_3114_MOESM1_ESM.docx]

Supplementary Material

| **Supplementary table 1. Microbiological documentation depending on the sample type (n=159).** | | | | | |
| --- | --- | --- | --- | --- | --- |
|  | Overall  n=159 | Sputum  n=33 | ETA  n=71 | BAL  n=34 | BBS  n=21 |
| *Escherichia coli* | 17 (11) | 2 (6) | 5 (7) | 6 (18) | 4 (19) |
| *Pseudomonas aeruginosa* | 15 (9) | 1 | 7 (10) | 2 (6) | 5 (24) |
| *Staphylococcus aureus* | 15 (9) | - | 5 (7) | 6 (18) | 4 (19) |
| *Klebsiella pneumoniae groupe* | 10 (6) | 1 | 6 (8) | 2 (6) | 1 |
| *Haemophilus influenzae* | 10 (6) | 3 (9) | 3 (4) | 2 (6) | 2 (9) |
| *Streptococcus pneumoniae* | 6 (4) | - | 3 (4) | 2 (6) | 1 |
| *Enterobacter cloacae complex* | 7 (4) | - | 5 (7) | 1 (3) | 1 (5) |
| *Enterobacter aerogenes* | 4 (3) | 1 | 2 (3) | - | 1 (5) |
| Citrobacter freundii | 2 (1) | - | 2 (3) | - | - |
| *Serratia marcescens* | 2 (1) | - | - | - | 2 (9) |
| *Morganella morganii* | 2 (1) | - | - | 1 | 1 |
| *Raoultella* | 2 (1) | - | 1 | 1 | - |
| *Stenotrophomonas maltophilia* | 2 (1) | - | - | - | 2 (9) |
| *Acinetobacter calcoaceticus-baumanii complex* | 1 | - | 1 | - | - |
| *Legionella pneumophila* | 1 | - | 1 | - | - |
| Results are presented as n (%)  Microbiological documentation is obtained through culture for all pathogens presented, apart from *Legionella pneumophila* which was detected using molecular techniques*.* Only pathogens cultured at pre-defined levels are presented (≥10^3^ CFU/ml for blind bronchial sampling, ≥10^4^ CFU/ml for bronchoalveolar lavage, ≥10^5^ CFU/ml for endotracheal aspiration, and ≥10^7^ CFU/ml for sputum samples). ETA = endotracheal aspirate, BAL= bronchoalveolar lavage, BBS= blind bronchial sampling | | | | | |

| **Supplementary table 2. Impact of the rm-PCR results on antibiotic prescription, according to the multidisciplinary committee and regarding sample type (n=159).** | | | | | |
| --- | --- | --- | --- | --- | --- |
|  | Overall  n=159 | Sputum  n=33 | ETA  n=71 | BAL  n=34 | BBS  n=21 |
| Antibiotic modification | 123 (77) | 21 (64) | 58 (82) | 27 (80) | 17 (81) |
| De-escalation | 63 (40) | 8 (24) | 30 (40) | 17 (50) | 8 (38) |
| Escalation | 35 (22) | 8 (24) | 18 (25) | 4 (12) | 5 (24) |
| Undetermined | 25 (16) | 5 (15) | 10 (14) | 6 (18) | 4 (19) |
| No change | 36 (23) | 12 (36) | 13 (18) | 7 (21) | 4 (19) |
| Results are presented as n (%)  ETA = endotracheal aspirate, BAL= bronchoalveolar lavage, BBS= blind bronchial sampling | | | | | |

**Supplementary table 3. Description of inadequate antibiotic regimen.**

| Sample type | Routine empirical therapy^a^ | Results of rm-PCR  bin/mL | rm-PCR–guided^b^  therapy | Result of conventional techniques  UFC/mL |
| --- | --- | --- | --- | --- |
| **Both routine and rm-PCR therapies inadequate (n=9)** | | | | |
| ETA | Piperacillin-Tazobactam  + Aminoglycoside | 10^7^ *Enterobacter cloacae complex*  10^7^ *Pseudomonas aeruginosa* Rhinovirus/Enterovirus | Piperacillin-Tazobactam  + Aminoglycoside | 10^6^ AmpC-producing *Enterobacter cloacae complex*, intermediate to amikacin |
| BBS | None | 10^6^ *Staphylococcus aureus*  Rhinovirus/Enterovirus | Carbapenem  + Aminoglycoside | 10^6^ *Stenotrophomonas maltophilia*  10^2^ *MSSA* |
| BBS | Piperacillin-Tazobactam  + Aminoglycoside | 10^5^ *Staphylococcus aureus* | Cefazoline | 10^7^ *Stenotrophomonas maltophilia*  10^5^ *MSSA*  10^4^ *Candida* sp |
| ETA | Piperacillin-Tazobactam | 10^7^ *Pseudomonas aeruginosa*  10^7^ *Klebsiella pneumoniae*  10^7^ *Proteus* spp.  10^7^ *Enterobacter cloacae complex*  10^6^ *Escherichia coli* | Piperacillin-Tazobactam | 10^7^  *Pseudomonas aeruginosa* resistant to piperacillin-tazobactam  10^7^ *Klebsiella pneumoniae* intermediate to piperacillin-tazobactam |
| ETA | Cefepime  +Metronidazole | 10^6^ *Enterobacter cloacae complex*  10^6^ *Escherichia coli*  10^7^ *Klebsiella pneumoniae*  10^7^ *Proteus* spp.  10^7^ *Pseudomonas aeruginosa*  10^4^ *Staphylococcus aureus* | Piperacillin-Tazobactam | 10^7^ *Pseudomonas aeruginosa* resistant to all beta-lactams  10^6^ *Klebsiella pneumoniae* |
| ETA | Amoxicillin-Clavulanate | 10^7^  *Pseudomonas aeruginosa*  10^4^ *Escherichia coli*  CTX-M positive | Piperacillin-Tazobactam | 10^5^  *Pseudomonas aeruginosa* resistant to all beta-lactam except penems  10^4^ ESBL-producing *Escherichia coli* (piperacillin-tazobactam susceptible) |
| ETA | Cefotaxime | 10^7^ *Acinetobacter baumannii* complex  10^6^ *Pseudomonas aeruginosa*  10^5^ *Escherichia coli* | Piperacillin-Tazobactam | 10^6^*Acinetobacter baumannii* complex resistant to all beta-lactams  10^6^ *Escherichia coli* resistant to piperacillin-tazobactam |
| sputum sample | Piperacillin-Tazobactam  +Macrolide | 10^6^ *Klebsiella pneumoniae*  10^7^ *Pseudomonas aeruginosa*  10^6^ *Proteus* spp.  10^6^ *Staphylococcus aureus*  10^5^ *Serratia marcescens* | Piperacillin-Tazobactam | 10^6^ AmpC-*Klebsiella pneumoniae*  10^6^ *Proteus vulgaris*  10^6^ *MSSA* |
| **Routine therapy inadequate / rm-PCR therapy adequate (n=14)** | | | | |
| sputum sample | Amoxicillin | 10^7^ *Haemophilus influenzae* | Amoxicillin-Clavulanate | 10^7^ penicillinase producing-*Haemophilus influenzae*  10^2^ *Candida sp* |
| ETA | Amoxicillin-Clavulanate | 10^7^ *Enterobacter aerogenes*  10^7^ *Haemophilus influenzae* | Cefepime | 10^6^ *Enterobacter aerogenes*  10^6^ *Citrobacter koserii*  <10^5^ *Candida sp* |
| ETA | Amoxicillin | 10^7^ *Enterobacter cloacae complex*  10^5^ *Escherichia coli*  10^4^ *Klebsiella oxytoca* | Cefepime  +Metronidazole | 10^6^ *Enterobacter cloacae complex* |
| ETA | None | 10^7^ *Enterobacter cloacae complex* | Cefepime  +Metronidazole | 10^7^ AmpC-*Enterobacter cloacae complex*  10^7^ AmpC-*Citrobacter freundii* |
| BAL | Ceftazidime  + Aminoglycoside | 10^7^ *Staphyloccocus aureus*  10^5^ *Escherichia coli*  mecA/C and MREJ positive | Cefotaxime  +Vancomycin | 10^6^ *MRSA*  10^5^ *Escherichia coli* |
| BAL | None | Negative | Piperacillin-Tazobactam | 10^5^ Corynebacterium propinquum |
| ETA | Cefotaxime  +Metronidazole | 10^7^ *Klebsiella pneumoniae*  10^4^ *Klebsiella oxytoca*  10^7^ *Escherichia coli*  10^7^ *Enterobacter cloacae complex*  10^6^ *Enterobacter aerogenes*  CTXM + | Carbapenem | 10^7^ ESBL-producing *Escherichia coli*  10^7^ *Raoultella ornithinolytica*  10^3^*Candida albicans* |
| ETA | Cefotaxime  +Metronidazole  +Fluoroquinolone | 10^6^ *Staphyloccocus aureus*  mecA/C and MREJ positive | Linezolide | 10^4^ *MRSA*  *10^3^ Candida sp* |
| ETA | Amoxicillin-Clavulanate | 10^7^ *Enterobacter cloacae complex*  10^4^ *Haemophilus influenzae* | Cefotaxime | 10^4^*Enterobacter cloacae complex*  Adenovirus |
| ETA | Piperacillin-Tazobactam | 10^7^ *Pseudomonas aeruginosa*  10^6^ *Enterobacter cloacae complex*  CTX-M positive | Carbapenem | 10^6^*Pseudomonas aeruginosa* resistant to ticarcillin, susceptible to piperacillin, intermediate to meropenem  10^5^ ESBL-producing *Enterobacter cloacae complex*  10^5^*Candida albicans* |
| sputum sample | Fluoroquinolone | 10^7^ *Pseudomonas aeruginosa*  10^6^ *Haemophilus influenzae*  10^4^ *Enterobacter aerogenes*  CTX-M positive | Carbapenem | 10^7^ *Pseudomonas aeruginosa* resistant to all beta-lactams except penems and to fluoroquinolones  10^5^ *Stenotrophomonas maltophila*  10^5^ *Enterobacter aerogenes*  10^4^ *Candida albicans* |
| sputum sample | Amoxicillin-Clavulanate | *Mycoplasma pneumoniae* | Macrolide | *Mycoplasma pneumoniae* |
| sputum sample | Amoxicillin-Clavulanate | 10^5^ *Pseudomonas aeruginosa*  10^4^ *Haemophilus influenzae* | Piperacillin-Tazobactam | 10^6^ *Pseudomonas aeruginosa*  10^6^ *Candida albicans* |
| BAL | Amoxicillin-Clavulanate | 10^5^ *Enterobacter aerogenes* | Piperacillin-Tazobactam | 10^4^ *Enterobacter aerogenes* |
| **rm-PCR therapy inadequate / routine therapy adequate (n=4)** | | | | |
| BAL | Carbapenem  +Cotrimoxazole  +Daptomycin | none | none | 10^7^ *Morganella morganii* |
| BAL | Ceftolozane-tazobactam | 10^7^ *Pseudomonas aeruginosa* | Ceftazidime | 10^7^ *Pseudomonas aeruginosa* resistant to ceftazidime, susceptible to carbapenems and ceftolozane-tazobactam |
| ETA | Cefepime | 10^7^ Streptococcus pneumoniae  10^7^ *Klebsiella Pneumoniae*  10^7^ *Enterobacter aerogenes*  10^6^ *Escherichia coli*  10^5^ *Enterobacter cloacae complex* | Piperacillin-Tazobactam | 10^8^ *Streptococcus pneumoniae*  10^7^ *AmpC -Enterobacter aerogenes*  10^7^*Citrobacter freundii* |
| sputum sample | Cefepime | 10^7^ *Enterobacter cloacae complex*  10^4^ *Staphyloccocus aureus* | Piperacillin-Tazobactam | 10^5^ *AmpC -Enterobacter cloacae complex* |

rm-PCR= real time multiplex Polymerase Chain Reaction, MRSA=Methicillin-resistant Staphylococcus aureus, ESBL=Extended Spectrum Beta-lactamase, AmpC=overproduced AmpC β-lactamase, ETA=Endotracheal Aspiration, BAL=Bronchoalveolar Lavage, BBS = Blind Bronchial Sampling

Routine empirical therapy is the antibiotic regimen that was actually administered to the patient in routine care.

rm-PCR-guided therapy is the antibiotic regimen proposed by the multidisciplinary committee based on clinical data and rm-PCR result.

Unless specified, detection of resistance genes by rm-PCR were negative.
